# Selective Senolysis of 5FU-Induced CRC Senescent Cells by Piceatannol Through Mitochondrial Depolarization and AIF-Dependent Apoptosis

**DOI:** 10.3390/ijms26189134

**Published:** 2025-09-18

**Authors:** Alessia Ambrosino, Deanira Patrone, Claudia Moriello, Sura Hilal Ahmed Al-Sammarraie, Ida Lettiero, Mauro Finicelli, Dario Siniscalco, Nicola Alessio

**Affiliations:** 1 Experimental Medicine Department, “Luigi Vanvitelli” Campania University, 80138 Naples, Italy; alessia.ambrosino@unicampania.it (A.A.); deanirantonietta.patrone@unicampania.it (D.P.); dario.siniscalco@unicampania.it (D.S.); 2 Department of Biotechnology College, Applied Science University of Samarra, Samarra 34010, Iraq; 3 Department of Medicine, Surgery and Dentistry, “Scuola Medica Salernitana”, University of Salerno, 84081 Baronissi, Italy; ilettiero@unisa.it; 4 Research Institute oh Terrestrial Ecosystems (IRET), National Research Council of Italy, 80128 Naples, Italy; mauro.finicelli@cnr.it

**Keywords:** senescence, Piceatannol, 5-Fluorouracil, apoptosis, mitochondria

## Abstract

Chemotherapy-induced senescence (CIS) contributes to tumor persistence and relapse. In this study, we investigated the senolytic activity of piceatannol (PCT) in 5-fluorouracil (5FU)-induced senescent colorectal cancer (CRC) cells. Senescence was established in P53-proficient HCT116 cells and normal colon fibroblasts (CCD18Co) following prolonged 5FU exposure, as shown by increased SA-β-gal activity, upregulation of P16, P21, and P53, mitochondrial depolarization, and enhanced oxidative stress. Subsequent PCT treatment selectively induced apoptosis in senescent populations, while non-senescent or p53-mutant, senescence-resistant HT29 cells were minimally affected. This effect was prevented by N-acetylcysteine, indicating a redox-sensitive mechanism. Mechanistically, PCT triggered mitochondrial depolarization and AIF-associated, caspase-independent apoptosis without increasing ROS. Morphological analysis with MitoTracker and quantitative morphometry using Fiji confirmed a fragmented mitochondrial network, characterized by reduced form factor, length, and number per cell. Western blotting revealed downregulation of fusion proteins (MFN1, MFN2), decreased FIS1, stable DRP1, and marked upregulation of the DRP1 adaptor MFF, consistent with suppressed fusion and enhanced fission competence. Together, these findings demonstrate that PCT selectively targets chemotherapy-induced senescent CRC cells through mitochondrial fragmentation and AIF-dependent apoptosis, highlighting its potential as an adjuvant strategy to limit the long-term burden of therapy-induced senescence.

## 1. Introduction

Chemotherapy remains a cornerstone in the treatment of solid tumors, including colorectal cancer [1]. Among the most widely used drugs, 5-Fluorouracil (5FU) is cytotoxic through inhibition of DNA synthesis and RNA metabolism. However, rather than inducing apoptosis in all tumor cells, 5FU frequently leads to a state of prolonged therapy-induced senescence (TIS), characterized by irreversible cell cycle arrest and the acquisition of a senescence-associated secretory phenotype (SASP) [2]. This phenotype is increasingly recognized for its dual role in cancer, potentially contributing to tumor relapse, inflammation, and resistance to further treatment [3,4].

The selective elimination of senescent cells using senolytic compounds has emerged as a promising therapeutic strategy in age-related disorders and, more recently, in oncology [5]. Senolytics are small molecules that can induce apoptosis selectively in senescent cells, proliferating, or quiescent populations [6]. While a number of senolytic drugs have been characterized, such as navitoclax, quercetin, and fisetin, there is growing interest in natural compounds with potential senolytic or senomorphic (SASP modulating) activity [7,8].

Piceatannol (PCT) is a natural polyphenol structurally related to resveratrol, found in grapes and other fruits, with known antioxidant and pro-apoptotic properties [9,10]. Compared to resveratrol, PCT exhibits a more complex metabolic profile, greater metabolic stability, and a better bioavailability profile, which may contribute to its distinct biological activities [11]. Previous studies have shown its ability to modulate mitochondrial ROS, alter Bcl-2 family protein expression, and enhance caspase activation [12,13]. However, its role as a senolytic agent in cancer models, particularly in cells rendered senescent by chemotherapy, remains unclear. Given the clinical concern that therapy-induced senescent cells contribute to tumor relapse, inflammation, and resistance to treatment, and the limited availability of natural compounds with well-characterized senolytic mechanisms, further investigation of PCT in this context is warranted.

In this study, we modeled chemotherapy-induced senescence using colorectal cancer cell lines (HCT116 and HT29) exposed to 5FU. Our objective was to evaluate whether PCT acts as a senolytic agent in this context. We show that PCT selectively induces apoptosis in 5FU-induced senescent HCT116 cells, largely through an AIF-associated, caspase-independent pathway, while sparing non-senescent or senescence-resistant cells such as HT29. These findings suggest that PCT has senolytic properties and reduces senescence markers in colorectal cancer models [14].

## 2. Results

### 2.1. 5FU Treatment Induces a Senescent Phenotype in Normal and Tumor Cells

To model therapy-induced senescence, we treated human colon fibroblasts (CCD18Co, CCD) and colorectal cancer cell lines (HCT116 and HT29) with 1 µM 5-Fluorouracil (5FU) for five days. This treatment induced a strong senescent phenotype, particularly in CCD and HCT116 cells, as evidenced by various senescence-associated markers.

**Figure 1:**
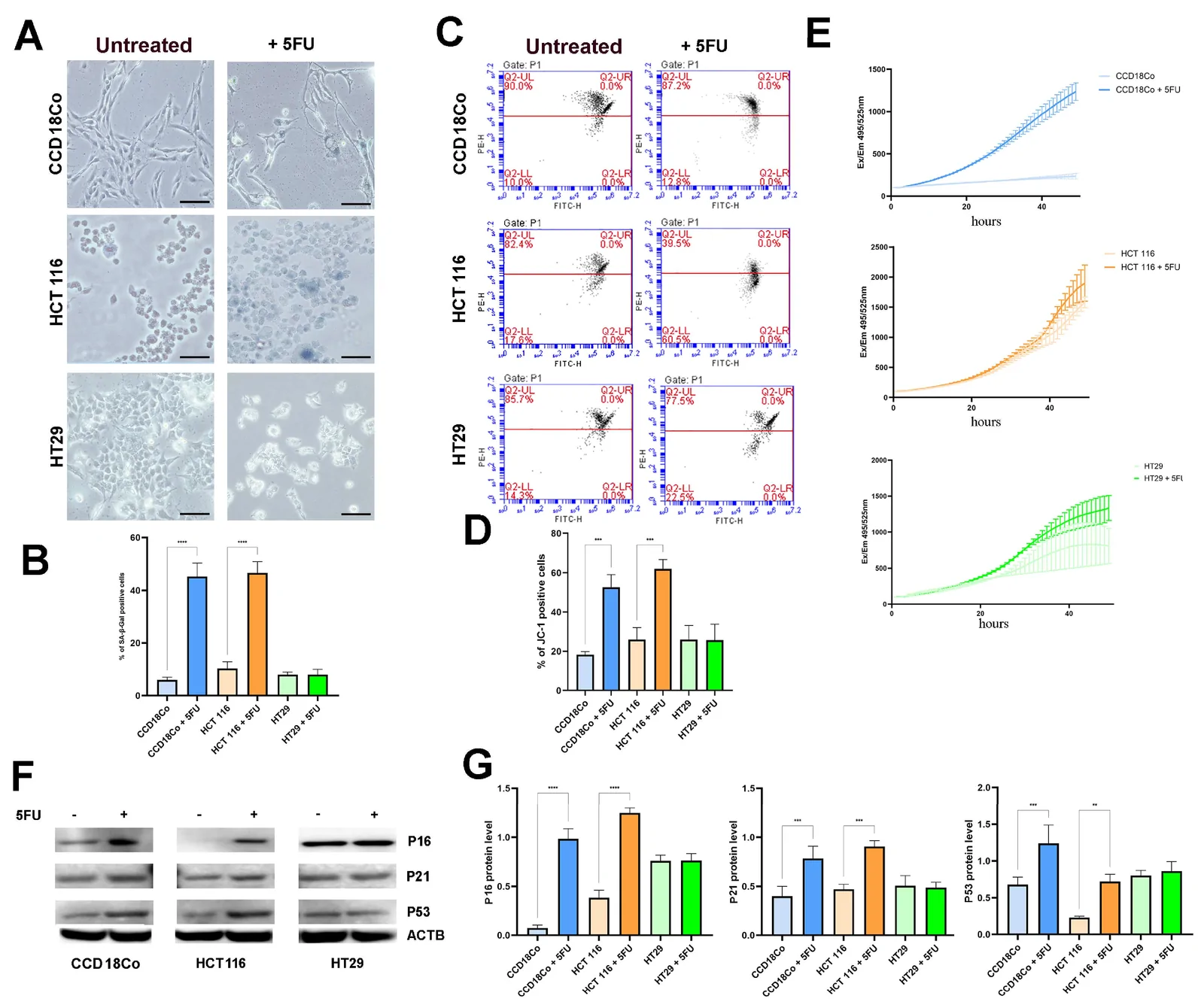
5FU induces senescence in normal and tumor cells. (**A**) Representative images of SA-β-galactosidase staining in CCD18Co, HCT116, and HT29 cells treated or not with 5FU (1 µM, 5 days). Images were acquired at 40× magnification; the dark bar in each panel represents the scale bar (100 μm). (**B**) Quantification of SA-β-gal positive cells shows increased senescence in CCD18Co and HCT116 after 5FU treatment, while HT29 cells show only a minimal response (n = 3, ±SD; **** *p* < 0.0001). (**C**) JC-1 analysis of mitochondrial membrane potential, showing increased mitochondrial depolarization and fragmentation following 5FU in CCD18Co and HCT116. (**D**) Quantification of JC-1 positive cells (green/red) further supports increased mitochondrial depolarization in CCD18Co and HCT116 after 5FU treatment (n = 3, ±SD; *** *p* < 0.001). (**E**) DCF-DA fluorescence measurements indicate elevated ROS levels following 5FU treatment. (**F**) Representative Western blots of senescence markers (P16, P21, and P53). (**G**) Densitometric quantification of Western blot bands confirms increased expression of senescence-associated proteins after 5FU (n = 3, ±SD; ** *p* < 0.01, *** *p* < 0.001, **** *p* < 0.0001).

Senescence was first assessed by SA-β-galactosidase staining (Figure 1A,B). In untreated CCDs, SA-β-gal positivity was ~16–18%, which increased to ~30–33% after 5FU exposure. A similar increase was observed in HCT116 cells. HT29 cells with mutant P53 and increased resistance to senescence exhibited minimal changes in SA-β-gal activity.

Mitochondrial depolarization and fragmentation, hallmarks of senescence, were confirmed by JC-1 staining (Figure 1C,D), with the proportion of low-potential (red/green) mitochondria rising from ~23% to ~66% in CCDs, and from ~26% to ~58% in HCT116 cells, post-5FU treatment. Oxidative stress also significantly increased, as shown by DCF-DA fluorescence (Figure 1E), confirming enhanced ROS production in both CCD and HCT116 cells.

Western blotting also confirmed the senescence phenotype, showing upregulation of P16, P21, and P53 after 5FU treatment in CCD and HCT116 cells (Figure 1F,G). Together, these data validate the establishment of a therapy-induced senescence model in normal and tumor cells with functional P53.

### 2.2. Piceatannol Selectively Eliminates 5FU-Induced Senescent Cells

**Figure 2:**
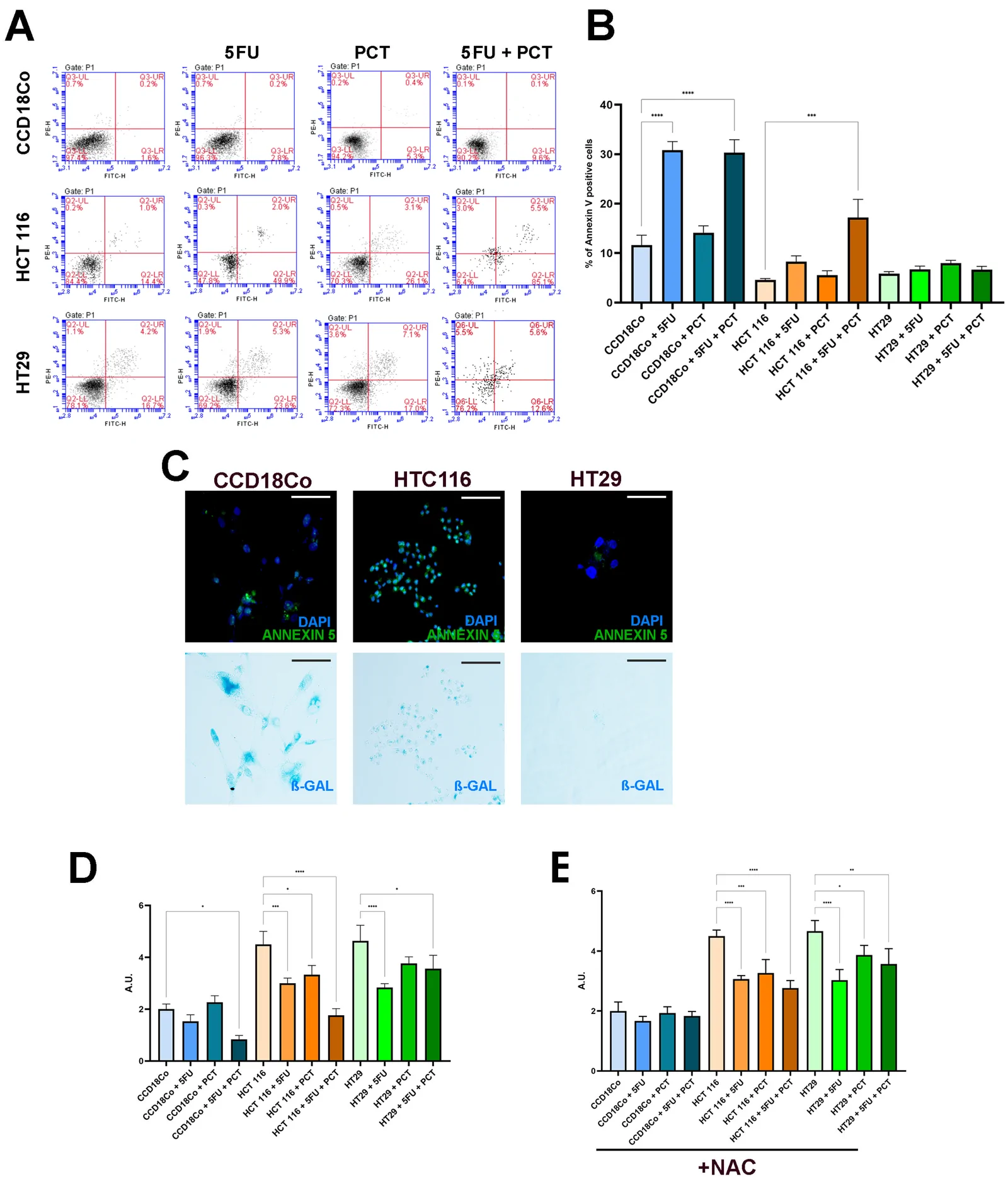
PCT selectively induces apoptosis in senescent cells. (**A**) Annexin V staining showed increased early apoptosis in CCD18Co and HCT116 cells treated with PCT (20 µM, 48 h), particularly in 5FU cell population treatment. (**B**) Quantification of apoptotic cell populations by flow cytometry (n = 3, ±SD; *** *p* < 0.001, **** *p* < 0.0001). (**C**) Immunocytochemical co-staining of SA-β-gal and Annexin V confirms that most apoptotic cells are also SA-β-gal positive, indicating selective elimination of senescent cells. Images were acquired at 40× magnification; the dark bar in each panel represents the scale bar (100 μm). (**D**) CCK8 viability assay shows that PCT significantly reduces viability in 5FU-primed CCD18Co and HCT116 cells (n = 3, ±SD; * *p* < 0.05, *** *p* < 0.001, **** *p* < 0.0001). (**E**) CCK8 viability assay in the presence of NAC. Co-treatment with NAC reverses the PCT-induced viability loss, indicating a ROS-dependent effect (n = 3, ±SD; * *p* < 0.05, ** *p* < 0.01, *** *p* < 0.001, **** *p* < 0.0001).

To evaluate senolytic activity, cells were treated with PCT (20 µM for 48 h) following 5FU exposure. Annexin V staining revealed a significant increase in early apoptosis in CCD and HCT116 cells that had been rendered senescent by 5FU, compared to untreated or 5FU-only controls (Figure 2A,B). Immunocytochemical co-staining with SA-β -galactosidase confirmed that apoptotic cells were predominantly senescent, indicating that PCT selectively targets the senescent population for elimination (Figure 2C).

Cell viability assays (CCK8) supported these data, with a significant reduction in viability in 5FU-pretreated CCD and HCT116 cells following PCT treatment (Figure 2D). In contrast, non-senescent populations and HT29 cells, which do not strongly undergo senescence, were minimally affected, supporting a selective senolytic mechanism of action. Importantly, the cytotoxicity of PCT was reversed by co-treatment with N-acetylcysteine (NAC), indicating that baseline oxidative tone plays a permissive role in PCT-mediated cell death (Figure 2E).

### 2.3. PCT Induces AIF-Dependent Apoptosis Without Increasing ROS

**Figure 3:**
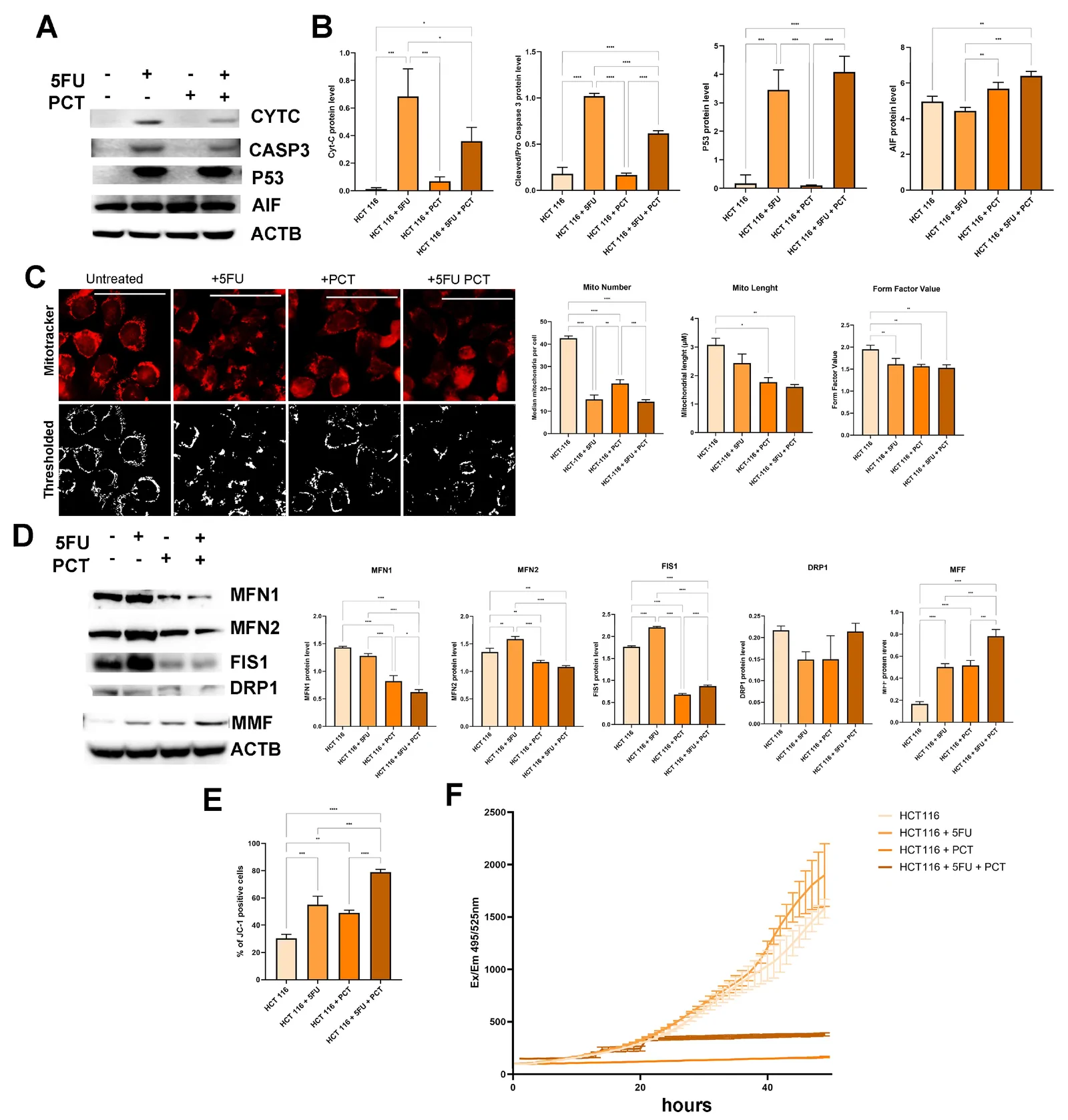
PCT induces mitochondrial depolarization and fragmentation and AIF-dependent apoptosis. (**A**) Western blots for cleaved caspase-3 (CASP3), cytochrome c (CYTC), and AIF in HCT116 cells following the indicated treatments. (**B**) Densitometric quantification shows that AIF increases with PCT treatment, whereas CASP3 and CYTC levels do not follow the same trend, suggesting a caspase-independent apoptotic mechanism (n = 3, ±SD; * *p* < 0.05, ** *p* < 0.01, *** *p* < 0.001, **** *p* < 0.0001). (**C**) Representative fluorescence micrographs of HCT116 cells stained with MitoTracker Green under the indicated conditions (untreated, 5FU, PCT, 5FU + PCT). The second row shows thresholded images processed with the MiNA plugin of Fiji to visualize mitochondrial structures. Quantitative analysis confirmed that the number of mitochondria per nucleus, mean form factor, and mean mitochondrial length were all reduced by 5FU and further decreased after combined 5FU + PCT treatment. Data are mean ± SEM (n ≥ 100 cells per condition, three independent experiments). Images were acquired at 100× magnification; the dark bar in each panel represents the scale bar (100 µm) (n = 3, ±SD; * *p* < 0.05, ** *p* < 0.01, *** *p* < 0.001, **** *p* < 0.0001). (**D**) Western blot analysis of mitochondrial dynamics proteins in HCT116 cells under the indicated conditions. MFN1 and MFN2 were downregulated by PCT; FIS1 also decreased, while DRP1 remained stable. The DRP1 adaptor MFF was upregulated by 5FU and strongly increased in the 5FU + PCT condition. β-actin was used as a loading control. Right panels: densitometric quantification normalized (n = 3, ±SD; * *p* < 0.05, ** *p* < 0.01, *** *p* < 0.001, **** *p* < 0.0001). (**E**) Quantification of JC-1 (green/red) fluorescence shows increased mitochondrial depolarization in senescent cells treated with PCT (n = 3, ±SD; ** *p* < 0.01, *** *p* < 0.001, **** *p* < 0.0001). (**F**) DCF-DA fluorescence analysis reveals reduced ROS levels in 5FU-primed cells treated with PCT.

To explore the mechanism behind PCT-induced senolysis, we evaluated mitochondrial function and cell death pathways. Western blot analysis showed that cleaved caspase-3 levels were not elevated in 5FU + PCT conditions compared to 5FU alone (Figure 3A,B), likely due to apoptotic cell loss at harvest. Cytochrome c levels were also relatively unchanged across conditions. In contrast, AIF levels increased notably in HCT116 cells treated with both 5FU and PCT, suggesting a caspase-independent, mitochondria-mediated apoptotic pathway. Consistently, MitoTracker imaging revealed marked mitochondrial fragmentation in 5FU + PCT–treated HCT116 cells, with a punctate distribution compared to the filamentous morphology observed in untreated or 5FU-only cells (Figure 3C).

To better understand these changes, we examined proteins regulating mitochondrial dynamics. Western blot analysis demonstrated that PCT reduced MFN1 and MFN2, while DRP1 remained unchanged, and FIS1 decreased (Figure 3D). Notably, the DRP1 adaptor MFF was upregulated by 5FU and strongly increased in the 5FU + PCT condition. These results indicate that PCT does not act by elevating DRP1 abundance but instead shifts the balance of mitochondrial dynamics by suppressing fusion (↓ MFN1/2) and enhancing fission competence via MFF upregulation, thereby promoting mitochondrial fragmentation.

JC-1 analysis showed enhanced mitochondrial depolarization in the senescent populations treated with PCT, particularly in the HCT116 + 5FU group (Figure 3E), aligning with AIF activation and loss of mitochondrial integrity. Consistently, MitoTracker imaging revealed marked mitochondrial fragmentation in 5FU + PCT-treated HCT116 cells, with a punctate distribution compared to the filamentous morphology observed in untreated or 5FU-only cells (Figure 3C). Quantitative analysis of mitochondrial morphology using Fiji confirmed these observations: form factor (a measure of circularity and branching), mean mitochondrial length, and the number of mitochondria per cell all significantly decreased in 5FU-treated cells, with the most pronounced reductions observed in the 5FU + PCT group (Figure 3C).

In contrast to what is typically observed with pro-apoptotic treatments, PCT did not elevate ROS levels. Indeed, DCF-DA fluorescence showed that ROS decreased over time in HCT116 + 5FU + PCT cells, compared to 5FU alone (Figure 3F), suggesting that PCT-induced apoptosis is not dependent on ROS increase, but may still rely on a basal oxidative state, as evidenced by the NAC protection experiments.

### 2.4. PCT Promotes Long-Term Resolution of the Senescent Phenotype

**Figure 4:**
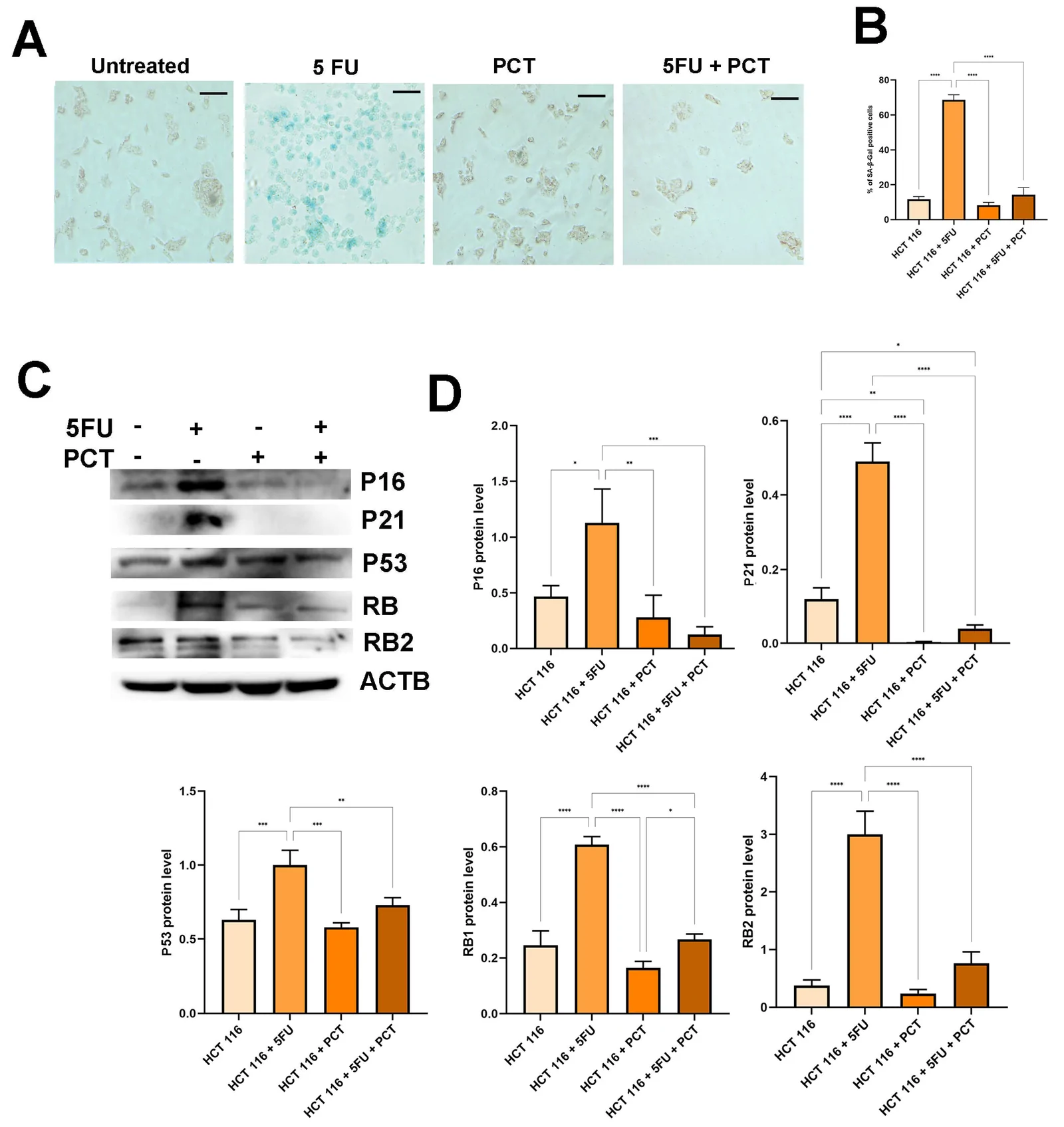
Long-term reduction in senescence markers after PCT treatment. (**A**) Representative SA-β-galactosidase staining images taken 7 days after PCT treatment of HCT116 cells. Images were acquired at 40× magnification; the dark bar in each panel represents the scale bar (100 μm). (**B**) Quantification of SA-β-gal positive cells demonstrates a significant reduction in senescence after PCT compared to the 5FU only group (n = 3, ±SD; **** *p* < 0.0001). (**C**) Western blot showing P16, P21, P53, RB, and RB2 expression at day 7. (**D**) Densitometric analysis confirms decreased expression of all tested senescence markers following PCT (n = 3, ±SD; * *p* < 0.05, ** *p* < 0.01, *** *p* < 0.001, **** *p* < 0.0001).

To find the fate of the surviving cells, we evaluated senescence markers seven days after PCT treatment. SA-β-galactosidase staining showed a clear reduction in senescent cell frequency compared to 5FU-only groups (Figure 4A,B). Western blot analysis confirmed this, with lower levels of P16, P21, P53, RB, and RB2 (Figure 4C,D), indicating that PCT not only eliminates senescent cells but may also promote the resolution of senescence in residual populations.

## 3. Discussion

The growing recognition that chemotherapy can induce a persistent senescent phenotype in cancer cells has raised concern about its long-term consequences, such as therapy resistance and tumor relapse. 5-Fluorouracil (5FU) [15,16], a frequently used chemotherapeutic agent, is known to induce DNA damage-associated senescence in P53-wild-type tumor cells, such as HCT116, as demonstrated in our study and in prior work [17,18]. On the contrary, P53-mutant HT29 cells, which poorly activate canonical senescence pathways, exhibited reduced senescence features following 5FU treatment, underscoring the importance of P53 status in the establishment of therapy-induced senescence (TIS) [19].

We focused our attention on PCT, a natural stilbene with previously described anti-inflammatory, antioxidant, and pro-apoptotic effects [10,20]. In this work, we show that PCT exhibits selective cytotoxicity against 5FU-induced senescent tumor cells, suggesting a senolytic effect. This is confirmed by Annexin V assays with SA-β-galactosidase staining, which showed that cells undergoing apoptosis were largely SA-β-gal positive, consistent with senescent cell-targeted killing. These findings align with previous reports of PCT being a senolytic compound in oxidative stress-induced models [7].

Contrary to the mechanism of action described for several senolytics, PCT did not increase mitochondrial ROS in senescent HCT116 cells. On the contrary, DCF-DA fluorescence levels were consistently reduced in 5FU-treated HCT116 exposed to PCT, suggesting that PCT pro-apoptotic activity in this setting may be ROS-independent. This characteristic is a distinct mechanism of senolysis, possibly through mitochondrial destabilization and AIF-mediated apoptosis, without exacerbating oxidative stress [21]. These features may be useful in avoiding off-target toxicity often associated with ROS-inducing senolytics [22].

We also observed that PCT reduced viability to some extent in non-senescent HCT116 cells. This mild cytotoxic effect may be related to basal oxidative stress levels or off-target activity at the concentration used. However, the effect was markedly more pronounced in 5-FU-induced senescent populations, consistent with selective senolysis.

Interestingly, Western blot analysis showed that cleaved caspase-3 levels were not significantly elevated in 5FU + PCT-treated HCT116 cells, while AIF expression was markedly increased. This may indicate that PCT induces a caspase-independent form of apoptosis in senescent tumor cells, possibly via mitochondrial outer membrane permeabilization and AIF nuclear translocation [23]. The relatively stable levels of cytochrome c further support a non-canonical apoptotic mechanism. Notably, Apoptosis-Inducing Factor (AIF), which is released from mitochondria, can translocate to the nucleus and trigger DNA fragmentation, a mechanism also observed with other senolytic agents [24]. This nuclear translocation of AIF may represent a conserved pathway of caspase-independent cell death in senescence targeting strategies, as previously demonstrated [24].

In addition to JC-1 depolarization, morphological analysis with MitoTracker staining revealed that PCT treatment induced a striking shift from elongated, filamentous mitochondria to a fragmented, punctate network in 5FU-senescent HCT116 cells. This observation was substantiated by Western blot analysis of mitochondrial dynamics proteins, which showed that PCT reduced the fusion markers MFN1 and MFN2, while DRP1 levels remained unchanged, and FIS1 was decreased. Importantly, the DRP1 adaptor MFF was strongly upregulated [25], particularly in the 5FU + PCT condition. Taken together, these findings suggest that PCT does not enhance fission by increasing DRP1 abundance, but rather by suppressing fusion capacity and upregulating MFF, thereby favoring DRP1-mediated mitochondrial fragmentation. Such fragmentation is mechanistically linked to mitochondrial depolarization and AIF release, reinforcing our model of a caspase-independent, mitochondria-driven apoptotic pathway in senescent cells. Consistently, our quantitative analysis of mitochondrial morphology using Fiji confirmed this remodeling. Form factor, mitochondrial length, and number per cell were all reduced following 5FU treatment, and these decreases were markedly accentuated in the 5FU + PCT condition. These results demonstrate that PCT amplifies senescence-associated mitochondrial alterations, driving a fragmented mitochondrial phenotype that aligns with impaired fusion capacity (↓ MFN1/2), increased MFF expression, and enhanced susceptibility to caspase-independent apoptosis.

A potential limitation of our interpretation is the role of N-acetylcysteine (NAC) in shaping PCT’s mechanism of action. In our experiments, NAC rescued the PCT-induced loss of viability in 5FU-senescent HCT116 and CCD cells, even though PCT did not increase overall ROS levels and, indeed, reduced DCF-DA fluorescence over time. This suggests that PCT-induced cell death does not require an acute ROS surge but depends on a permissive redox tone established by 5FU. Mechanistically, NAC enhances glutathione synthesis and reduces thiol oxidation, which may stabilize mitochondrial permeability, prevent depolarization detected with JC-1, and limit AIF release and nuclear translocation [26]. NAC may also directly quench electrophilic metabolites of PCT, thereby attenuating its cytotoxic activity [27]. These findings support our model of a redox-sensitive, AIF-associated, caspase-independent apoptotic pathway, rather than a ROS-driven toxicity. However, we note the limitations of the assays used: DCF-DA primarily detects cytosolic peroxides and may underestimate compartment-specific ROS, while NAC itself can lower the DCF signal by enhancing antioxidant capacity. Moreover, reduced Annexin V/PI staining in the presence of NAC could reflect both decreased apoptosis and reduced tic membrane damage. Together, these considerations highlight the importance of ROS homeostasis in PCT-induced senolysis and suggest that additional assays—such as glutathione redox measurements, mitochondrial superoxide probes, or markers of necroptosis—will be needed to refine our mechanistic model.

Furthermore, we observed that PCT treatment not only eliminated senescent cells but also induced a persistent reduction in senescence markers in the surviving cell population. Western blot analysis performed 7 days after PCT treatment showed a significant reduction in RB, RB2, P53, P21, and P16 levels, associated with a reduction in SA-SA-β-Gal staining. This suggested that PCT may act as a senolytic agent and reduce senescence-associated markers. However, we did not directly assess SASP modulation, and further studies will be required to evaluate potential senomorphic effects [28].

Our findings also demonstrate that the co-treatment with the antioxidant N-acetylcysteine (NAC) suppressed the effects of PCT, both by inhibiting ROS generation and reducing viability [29]. These findings indicate that PCT-induced cell death is redox-sensitive rather than driven by an acute ROS increase. NAC likely stabilizes the redox tone and mitochondrial integrity, thereby limiting AIF release and apoptosis.

Future studies will be needed to determine whether PCT-mediated clearance of senescent tumor cells can effectively reduce the risk of colon cancer relapse.

In summary, our study supports a model in which PCT induces chemotherapy-induced senescent tumor cells, particularly in a P53-proficient context, through a ROS-mediated, AIF-dependent apoptotic pathway. These findings contribute to the growing field of senotherapy, where the combination of senescence-inducing drugs and senolytics may provide improved long-term therapeutic efficacy and limit recurrence.

## 4. Materials and Methods

### 4.1. Cell Culture and Reagents

Human colon fibroblast (CCD18Co) was cultured in MEM supplemented with 1% Non-essential amino acid, 10% fetal bovine serum, and 1% penicillin-streptomycin. HT29 (P53 mutant) and HCT116 (P53 wild-type) colorectal carcinoma cells were cultured in DMEM supplemented with 10% fetal bovine serum (FBS) and 1% penicillin-streptomycin. All cell cultures were maintained at 37 °C in a humidified 5% CO_2_ atmosphere.

5-Fluorouracil (5FU) was purchased from Sigma-Aldrich (St. Louis, MO, USA) and used at a final concentration of 1 µM for senescence induction. Piceatannol (PCT) was obtained from Selleck Chemicals (Houston, TX, USA) and dissolved in DMSO; the final treatment concentration was 20 µM.

Where indicated, cells were co-treated with N-acetylcysteine (NAC, 5 mM; Sigma-Aldrich).

### 4.2. Senescence Induction and Treatment Schedule

Cells were exposed to 1 µM 5FU for 5 consecutive days. After 24 h of recovery in drug-free medium, cells were treated with 20 µM PCT for an additional 48 h. Control cells were treated with vehicle (DMSO) under identical conditions. In some experiments, NAC was added simultaneously with PCT. Cells were seeded at equal densities for all experimental groups. The lower density observed in some conditions reflects differential cell growth and survival after 5FU exposure, rather than differences in initial seeding or treatment timing.

### 4.3. SA-β-galactosidase Staining

Senescent cells were identified using the Senescence SA-β-galactosidase Staining Kit (Cell Signaling Technology, Danvers, MA, USA) following the manufacturer’s protocol. Cells were fixed and stained, and blue-stained cells were quantified under a bright-field microscope in at least five random fields. Results are expressed as the percentage of positive cells relative to total nuclei.

### 4.4. Immunocytochemistry

For co-localization studies, cells were fixed and incubated with FITC-conjugated Annexin V (Elabscience, Houston, TX, USA) and SA-β-galactosidase, followed by appropriate fluorescent secondary antibodies. Nuclei were counterstained with DAPI. Images were acquired using a fluorescence microscope and analyzed with ImageJ software (Version: 2.16.0/1.54p).

### 4.5. Apoptosis Assay (Annexin V/PI)

Apoptosis was evaluated by flow cytometry using the Annexin V-FITC/Propidium Iodide Apoptosis Detection Kit (BioLegend, San Diego, CA, USA). Cells were collected, washed, and stained according to the manufacturer’s instructions. Quantification of early and late apoptotic populations was performed using a BD Accuri C6 cytometer.

### 4.6. Mitochondrial Membrane Potential (JC-1)

Mitochondrial integrity was assessed with the JC-1 Mitochondrial Membrane Potential Assay Kit (Thermo Fisher, Waltham, MA, USA). Cells were stained with JC-1 dye and analyzed by flow cytometry. Red/green fluorescence ratio was used as an indicator of mitochondrial polarization.

### 4.7. Reactive Oxygen Species Measurement (DCF-DA)

Intracellular ROS levels were measured using 2′,7′-dichlorodihydrofluorescein diacetate (DCF-DA; Sigma-Aldrich). Cells were incubated with 10 µM DCF-DA, and fluorescence (excitation 485 nm, emission 535 nm) was continuously monitored for 48 h at 37 °C using a CO_2_-independent medium in a GloMax microplate reader (Promega, Madison, WI, USA).

### 4.8. Cell Viability (CCK-8 Assay)

Cell proliferation and viability were measured with the Cell Counting Kit-8 (CCK-8; Dojindo, Mashiki-machi, Kumamoto, Japan). Cells were seeded in 96-well plates and treated as indicated. After treatment, 10 µL of CCK-8 reagent was added to each well and incubated for 2 h. Absorbance was measured at 450 nm using a microplate reader (Promega, Madison, WI, USA).

### 4.9. Mitochondrial Morphology Analysis

Mitochondrial morphology was assessed using MitoTracker Red (Thermo Fisher, Waltham, MA, USA). Cells were incubated with 100 nM dye for 30 min at 37 °C, washed, and imaged live using a fluorescence microscope (×100 oil immersion). Images were processed with Mitotraker analysis plugin of Fiji (Version: 2.16.0/1.54p). Representative images are shown with consistent exposure and scaling.

### 4.10. Western Blot Analysis

Whole cells were lysed in Lysis buffer supplemented with protease and phosphatase inhibitors. Protein concentration was determined using the Bradford assay. Equal amounts of protein were separated by SDS-PAGE and transferred to PVDF membranes. Blots were probed with primary antibodies against RB (#9309, CellSignaling, Danvers, MA, USA), RB2 (610262, BD, Franklin Lakes, NJ, USA), P53 (sc-126, SantaCruz Biotechnology, Dallas, TX, USA, P21 (sc-6246, SantaCruz), P16 (E-AB-65673, Elabscience, Houston, TX, USA), cleaved caspase-3 (CASP3, A13916, Antibodies, St. Louis, MO, USA), cytochrome c (CYTC, E-AB-64633, Elabscience, Houston, TX, USA), Apoptosis-Inducing Factor (AIF, MAS-15880, Invitrogen, Carlsbad, CA, USA), MFN1(E-AB-93135, Elabscience, Houston, TX, USA), MFN2 (E-AB-32025, Elabscience, Houston, TX, USA), FIS1(E-AB-67035, Elabscience, Houston, TX, USA), DRP1(E-AB.-93308, Elabscience, Houston, TX, USA), MFF (ab129075, abcam, Cambridge, UK), and ACTB (sc-47778, SantaCruz Biotechnology, Dallas, TX, USA), followed by HRP-conjugated secondary antibodies. Detection was performed using ECL substrate (Thermo Fisher, Waltham, MA, USA) and imaged with an Azure 300 system (Azure Biosystems, Dublin, CA, USA). The Red Ponceau was used as an internal control [15].

### 4.11. Statistical Analysis

All experiments were performed in triplicate unless otherwise stated. Data are expressed as mean ± SEM. Statistical significance was determined by one-way ANOVA with Tukey’s post-hoc test using GraphPad Prism 9. A *p*-value < 0.05 was considered statistically significant.

## 5. Conclusions

Our study demonstrates that 5-Fluorouracil induces a robust senescence program in both normal and P53-competent colorectal cancer cells, characterized by classical markers such as increased SA-β-galactosidase activity, mitochondrial depolarization, oxidative stress, AIF upregulation, and upregulation of P16, P21, and P53. Importantly, we show that PCT, a dietary polyphenol, exerts a senolytic effect by selectively inducing apoptosis in senescent cells, without significantly affecting non-senescent or senescence-resistant populations such as HT29 cells.

Mechanistically, PCT acts through mitochondrial depolarization and AIF-associated, caspase-independent apoptosis, with minimal involvement of ROS generation. This selective elimination of senescent cells is accompanied by a reduction in senescence markers among the surviving cell population, suggesting that PCT may also promote resolution of the senescent state in non-apoptotic cells.

Taken together, these findings support the use of PCT as a potential adjuvant to chemotherapy, capable of reducing the long-term burden of therapy-induced senescence and its associated detrimental effects. This action may help improve therapeutic outcomes and limit chronic damage in both tumor and normal tissues exposed to genotoxic stress.

## Data Availability

The original contributions presented in this study are included in the article. Further inquiries can be directed to the corresponding authors.
