# Correction: Ambrosino et al. Selective Senolysis of 5FU-Induced CRC Senescent Cells by Piceatannol Through Mitochondrial Depolarization and AIF-Dependent Apoptosis. *Int. J. Mol. Sci.* 2025, *26*, 9134

**DOI:** 10.3390/ijms27198751

**Published:** 2026-09-30

**Authors:** Alessia Ambrosino, Deanira Patrone, Claudia Moriello, Sura Hilal Ahmed Al-Sammarraie, Ida Lettiero, Mauro Finicelli, Dario Siniscalco, Nicola Alessio

**Affiliations:** 1 Experimental Medicine Department, “Luigi Vanvitelli” Campania University, 80138 Naples, Italy; alessia.ambrosino@unicampania.it (A.A.); deanirantonietta.patrone@unicampania.it (D.P.); dario.siniscalco@unicampania.it (D.S.); 2 Department of Biotechnology College, Applied Science University of Samarra, Samarra 34010, Iraq; 3 Department of Medicine, Surgery and Dentistry, “Scuola Medica Salernitana”, University of Salerno, 84081 Baronissi, Italy; ilettiero@unisa.it; 4 Research Institute oh Terrestrial Ecosystems (IRET), National Research Council of Italy, 80128 Naples, Italy; mauro.finicelli@cnr.it

In Figure 2A of the original publication [1], an inadvertent error occurred during the assembly of the figure. Specifically, two identical images were mistakenly inserted in place of the correct panels. The error was limited to the graphical representation of the figure and did not involve any changes to the underlying experimental data or quantitative analysis. The affected image has been replaced with the correct panel in the revised version of Figure 2A. This correction does not affect the results, interpretation, or conclusions of this study. No additional articles are associated with this correction. This correction was approved by the Academic Editor. The original publication has also been updated.

## Figures and Tables

**Figure 2:**
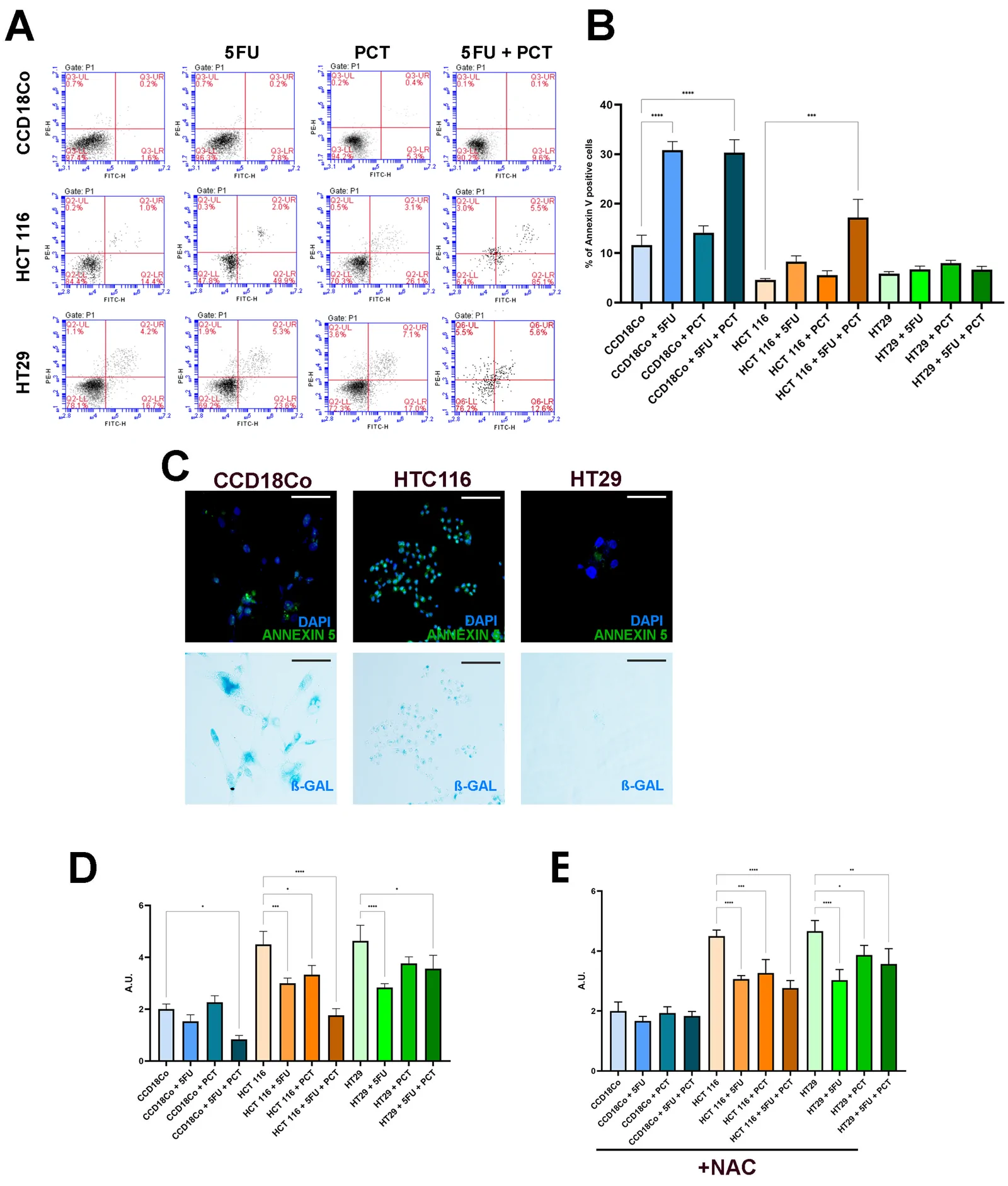
PCT selectively induces apoptosis in senescent cells. (**A**) Annexin V staining showed increased early apoptosis in CCD18Co and HCT116 cells treated with PCT (20 µM, 48 h), particularly in 5FU cell population treatment. (**B**) Quantification of apoptotic cell populations by flow cytometry (n = 3, ±SD; *** *p* < 0.001, **** *p* < 0.0001). (**C**) Immunocytochemical co-staining of SA-β-gal and Annexin V confirms that most apoptotic cells are also SA-β-gal positive, indicating selective elimination of senescent cells. Images were acquired at 40× magnification; the dark bar in each panel represents the scale bar (100 μm). (**D**) CCK8 viability assay shows that PCT significantly reduces viability in 5FU-primed CCD18Co and HCT116 cells (n = 3, ±SD; * *p* < 0.05, *** *p* < 0.001, **** *p* < 0.0001). (**E**) CCK8 viability assay in the presence of NAC. Co-treatment with NAC reverses the PCT-induced viability loss, indicating a ROS-dependent effect (n = 3, ±SD; * *p* < 0.05, ** *p* < 0.01, *** *p* < 0.001, **** *p* < 0.0001).
